# Venous Thrombosis Secondary to Acute Cytomegalovirus Infection in an Immunocompetent Host: Consideration for New Screening Guidelines

**DOI:** 10.7759/cureus.2742

**Published:** 2018-06-05

**Authors:** Sam Ngu, Naureen Narula, Talha N Jilani, Alexander Bershadskiy

**Affiliations:** 1 Internal Medicine, Staten Island University Hospital, Staten Island, USA; 2 Postgraduate, Ziauddin University; 3 Department of Hematology and Oncology, Staten Island University Hospital, Staten Island, USA

**Keywords:** cmv infection, screening, hematology, deep venous thrombosis

## Abstract

Serious thrombotic complications associated with an acute cytomegalovirus (CMV) infection in immunocompromised and immunocompetent patients are becoming increasingly recognized. While typically asymptomatic and self-limiting, an acute CMV infection appears to demonstrate a rare propensity for a vascular thrombosis, such as deep vein thrombosis (DVT), thrombophlebitis, and pulmonary embolism (PE). It remains unclear whether other predisposing factors play a role in its pathogenesis. We report the case of a young, immunocompetent male with extensive lower extremity DVT who was coincidentally found to be CMV-immunoglobulin M (IgM) seropositive. In light of the increasing prevalence of CMV-associated thrombotic events, we reviewed the current literature on its incidence, pathophysiology, clinical features, and thrombophilia screening to consider the possibility of CMV seropositivity as an independent risk factor for vascular events. This may have repercussions for screening guidelines and preventive strategies in those with active CMV infection.

## Introduction

Serious thrombotic complications associated with an acute cytomegalovirus (CMV) infection in immunocompromised and immunocompetent patients are becoming increasingly recognized. While typically asymptomatic and self-limiting in the latter, an acute CMV infection demonstrates a rare propensity for a vascular thrombosis, such as unprovoked deep vein thrombosis (DVT), superficial vein thrombophlebitis (SVTE), and venous thromboembolism (VTE). It remains unclear whether other predisposing factors play a role in its pathogenesis. The first reported cases were seen in immunocompromised patients, particularly human immunodeficiency virus (HIV)-seropositive and transplant patients on high-dose immunosuppressants who developed DVT and VTE [1]. Similar thrombotic events are increasingly recognized in immunocompetent individuals. It is suggested that CMV can reside within both superficial and deep arteries and veins, initiating endothelial and vascular inflammation that increases the propensity for thrombus formation. The predominant theory is that the infectious state transiently increases antiphospholipid antibodies (APLAs), which last until a resolution of symptoms. We report a case of a young, immunocompetent adult with acute CMV infection and no other pro-thrombotic risk factors, which was complicated by extensive bilateral lower extremity DVT and SVTE. In light of the increasing prevalence of CMV-associated thrombotic events in recent literature, we reviewed the incidence and pathophysiology to consider the possibility of CMV seropositivity as an independent risk factor for vascular events. This may have repercussions for screening guidelines and preventive strategies in those with an acute CMV infection.

## Case presentation

**Figure 1:**
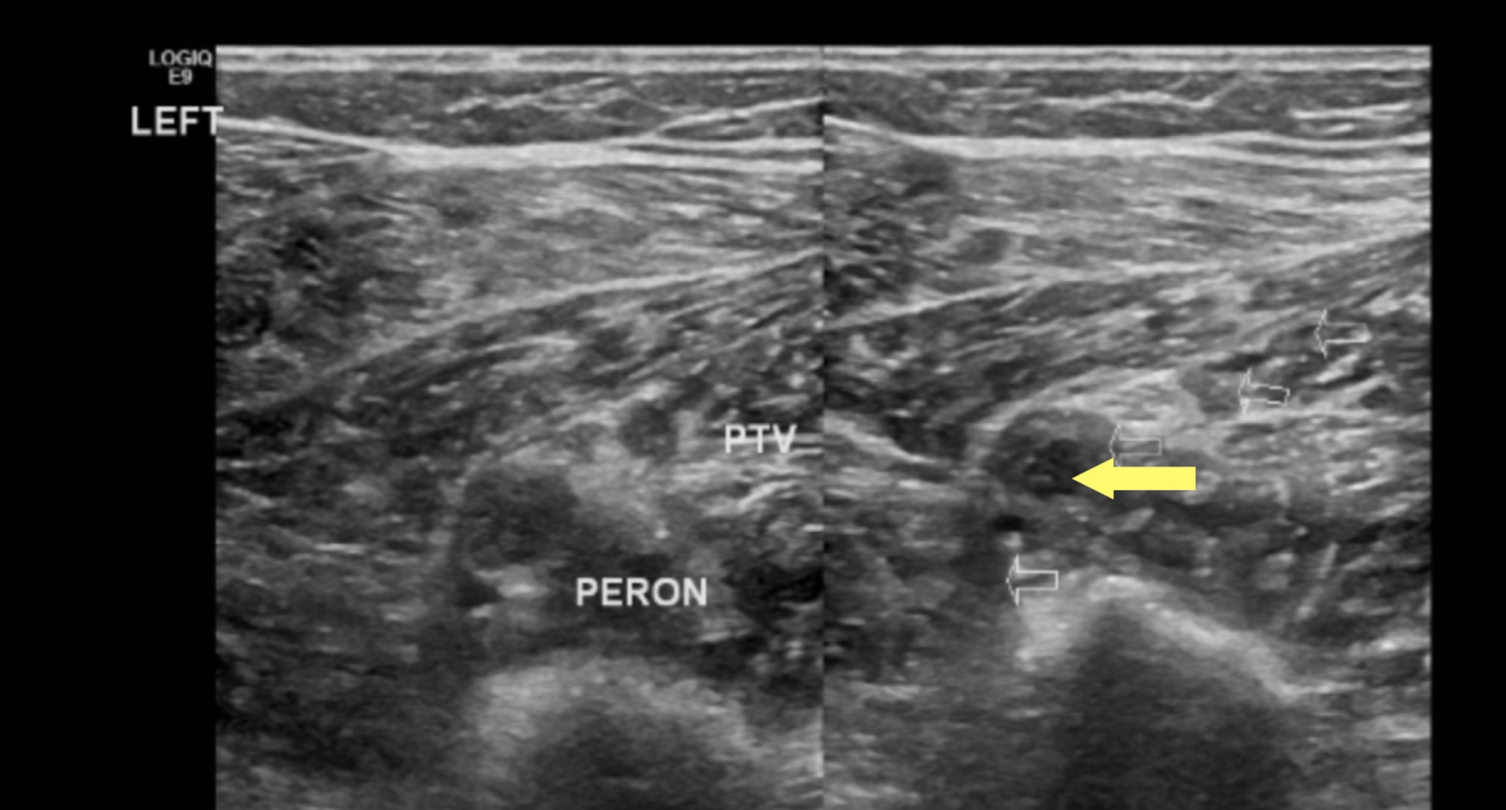
Left peroneal vein deep vein thrombosis (yellow arrow), axial view

**Figure 2:**
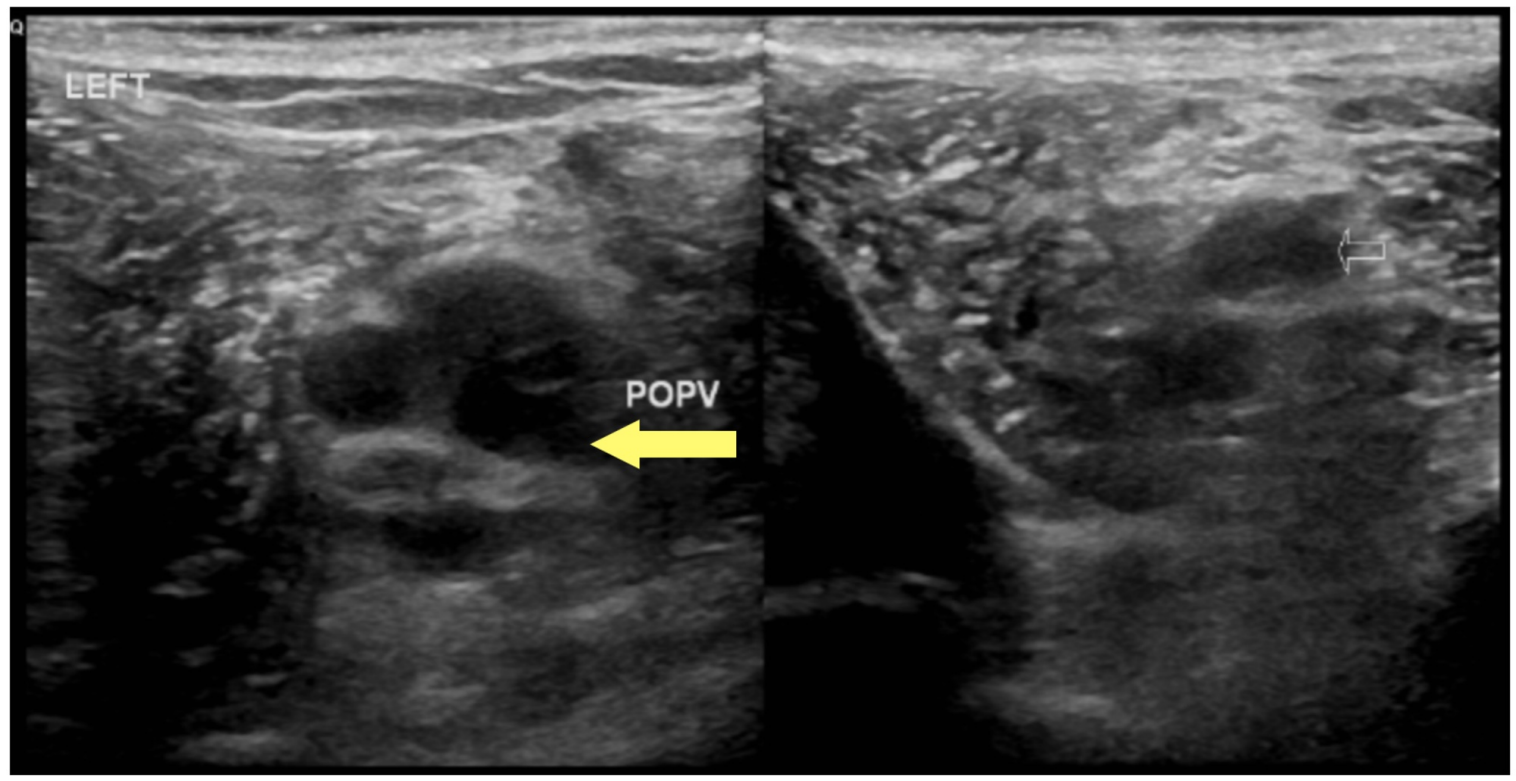
Left popliteal vein deep vein thrombosis (yellow arrow), axial view

**Figure 3:**
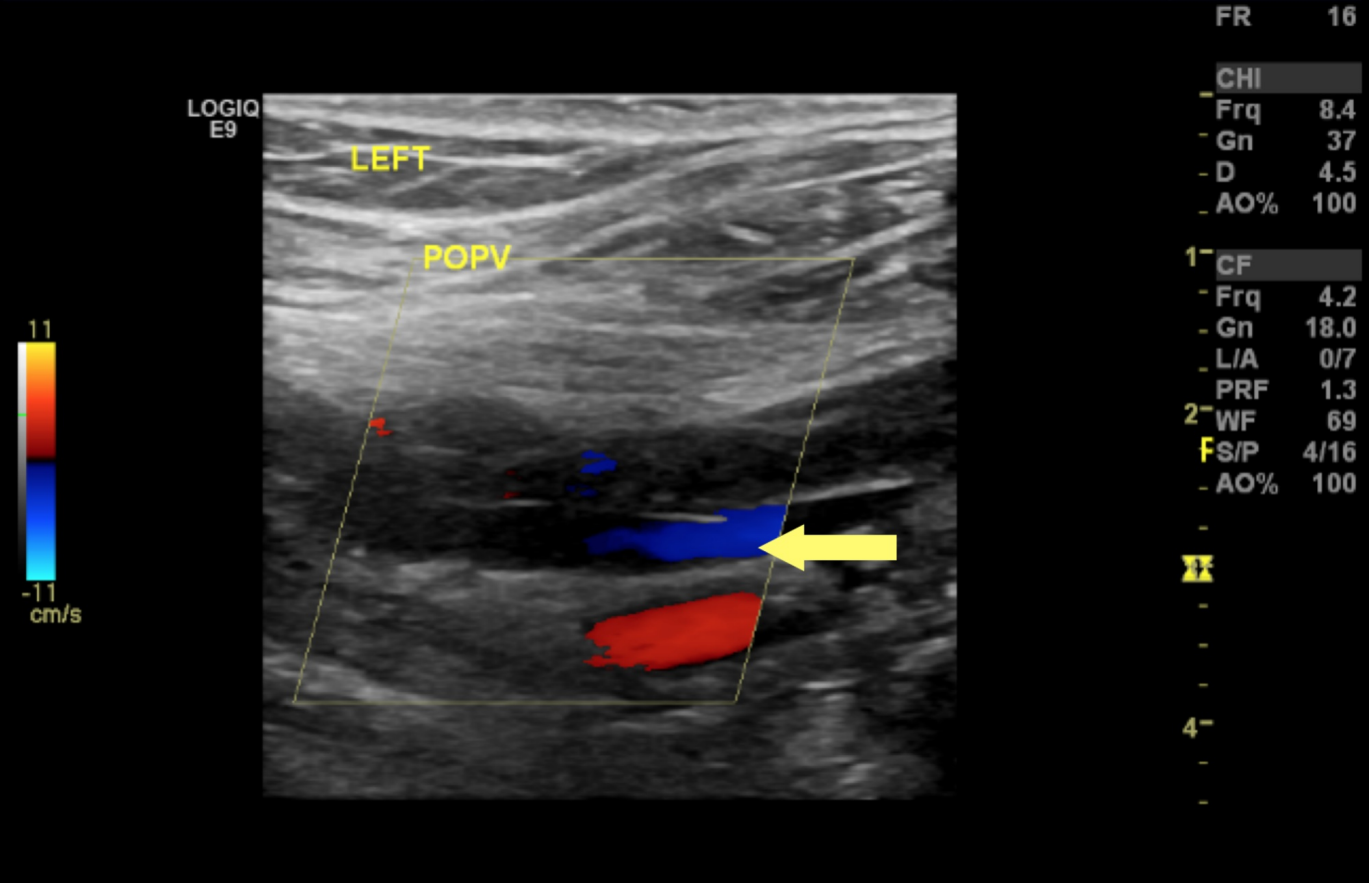
Doppler ultrasound for left popliteal vein showing partial occlusion

A previously healthy 27-year-old man presented with complaints of left calf pain and erythema of four days duration, which was preceded by a petechial rash of the bilateral lower extremities and left foot pain. He also reported low-grade fever (100.7 F) with associated chills. He was initially seen at a Level 1 trauma center where he underwent a Doppler investigation of the lower extremities with negative findings. Blood work at the time was reported normal. A worsening induration and swelling of the left lower extremity prompted him to seek further work-up. An inquiry into past medical and family history was non-contributory. He had a history of hernia repair and tonsillectomy. Social history was significant for recreational marijuana and cocaine use in the past. He was homosexual and reported being sexually active with one male partner and inconsistent contraception use. The patient denied weight loss, night sweats, recent travel, recent major illness or surgery, or steroid use. On admission, he was afebrile (98.8 F) and tachypneic (18 breaths per minute). A blood pressure of 132/75 mmHg, heart rate of 81 beats per minute, and oxygen saturation of 99% on room air were documented. The physical examination revealed a mildly enlarged spleen and confluent erythema of the bilateral lower extremities that were tender to touch. Blood work showed a normal white blood count of 7.33X10^9^/L, hemoglobin of 15.6 mg/dl with marked thrombocytopenia, and platelet count of 51X10^9^/L. An aspartate aminotransferase (AST) level of 289/L, alanine aminotransferase of 372/L, and direct bilirubin of 0.22 umol/L confirmed transaminitis. The D-Dimer level was 14,000 ng/ml. The venous duplex of the lower extremities showed extensive thrombosis in the left peroneal (Figure 1) and thrombosis of the left popliteal (Figures 2-3).

Computed tomography (CT) of the chest with contrast revealed borderline splenomegaly of size 13 cm. Extensive investigations eventually showed evidence of an active CMV infection with CMV-Immunoglobulin M (IgM) seropositivity (>240.0 AU/mL) and positive polymerase chain reaction (PCR). Serology was positive for the Epstein Barr virus (EBV) (368 U/mL). He received a platelet transfusion with symptomatic improvement. He was discharged home on Eliquis 5 mg twice daily; however, he returned to the hospital a week after with new symptoms. He reported right lower leg pressure-like pain that was exacerbated with walking. Doppler revealed right great saphenous vein superficial thrombophlebitis along with unchanged findings in the left leg. He was initially switched to Rivaroxaban 15 mg twice daily; and on clinical improvement, he was later discharged on Apixaban 5 mg twice daily.

## Discussion

An active CMV infection leading to vascular thrombosis was first documented in an immunocompetent host in 1984 [2]. Recognized primarily in immunocompromised patients, such as HIV and renal transplant recipients, CMV mononucleosis has been increasingly recognized as a thrombotic risk factor in immunocompetent patients. To the best of our knowledge to date, there are about 100 case reports, one retrospective case-control, two prospective studies, and one cohort trial reporting local thrombus formation of internal jugular veins, cerebral venous system, and splanchnic, ovarian, iliac, and branch retinal veins [3-7]. The two most common thrombotic events associated with CMV infections are lower-extremity DVT and/or VTE followed by a splanchnic vein thrombosis, such as portal vein thrombosis, superior mesenteric vein thrombosis, inferior mesenteric vein thrombosis, and colic vein thrombosis [1].

A CMV infection is common in the human population without gender predominance; infection is mainly subclinical and self-limiting in immunocompetent individuals. Seroprevalence rates range from 40%-100% depending on age, geographical location, and socioeconomic factors [8]. Those seriously affected are typically in extremes of age or have concomitant health conditions that weaken the immune system, presenting as fever, cervical lymphadenitis, and arthralgia. Pneumonia, myocarditis, pericarditis, colitis, and hemolytic anemia are less frequent occurrences. A retrospective study by Yildiz et al. suggested that VTE patients with synchronous acute CMV infection are comparatively younger (37.5 years vs 56.6 years, P = 0.0088) with female predominance (90% vs 56%; p = 0.026) [9].

Increasingly, studies in experimental mouse models and humans have suggested that CMV may play a role in the development of atherosclerosis through a persistent inflammation of the vascular system [10-12]. CMV DNA has been detected in atherosclerotic plaques and the presence of CMV correlates with restenosis in patients who have undergone coronary atherectomy or angioplasty [13-14]. A retrospective case-control study conducted in 2010, involving 140 subjects with acute CMV mononucleosis in a tertiary care center, found venous thrombosis in 6.4% of individuals with no thrombosis observed in matched control patients. The true incidence of thrombosis is likely higher since not every patient underwent thorough imaging studies to exclude thrombus formation. This study also reported that up to 65.7% of immunocompetent adults have either acquired or inherited predispositions for thrombosis [15]. Two prospective studies found that the prevalence of CMV seropositivity was higher in VTE patients [16-17].

The role of CMV in thrombogenesis is not yet known. The proposed mechanisms of pathogenesis include the promotion of platelet and leukocyte adhesion to the endothelium, factor X activation, smooth muscle proliferation, and increased production of thrombogenic factors, such as factor VIII, antiphospholipid antibody (APLA), and platelet-derived growth factor. Thrombotic events are usually limited to the venous system with cases of arterial thrombosis being exceptionally rare. The initiation of endothelial inflammation and vasculitis may explain the increased risk for occlusive vascular ischemia months to years after acquiring the infection [18]. The predominant theory is that the infectious state transiently increases APLAs, which disappear with the resolution of symptoms [2]. Another hypothesis is that the virus acquires procoagulant properties when CMV replicates inside endothelial cells. It has been shown to acquire procoagulant phospholipid (proPL) and tissue factor, which are necessary for thrombin generation [19]. Herpesviruses, including CMV and EBV, can additionally induce thrombogenesis during envelope formation by upregulating thrombin production and by facilitating the activation of factor X. Von Willebrand factor (vWF) expression is also known to increase over the course of the infection [20]. Reported cases support the use of anticoagulation with low-molecular-weight heparin and warfarin. While it has been thought that SVTE is a benign, self-limited disease, mounting evidence demonstrates an increased propensity to develop more serious thrombotic events. As a majority of CMV infections are subclinical in immunocompetent adults, its role in more subtle and seemingly pleomorphic effects are grossly underestimated. It remains to be seen whether the screening of all patients for CMV-IgM seropositivity with lower extremity Doppler will be cost-effective in reducing disease burden. Some recommend screening patients with CMV-IgM antibodies for those in which no obvious thrombosis risk factors were found.

## Conclusions

Due to its largely subclinical infection in immunocompetent adults, the role of CMV in vasculopathy and venous thrombosis has been underestimated. Patients with CMV-associated thrombosis benefit from the prompt administration of anticoagulants. Physicians should be cognizant of the association between acute viral infections, particularly CMV mononucleosis, in patients with idiopathic thromboses.
